# Fabrication and Characterization of a High-Performance Multi-Annular Backscattered Electron Detector for Desktop SEM †

**DOI:** 10.3390/s18093093

**Published:** 2018-09-14

**Authors:** Wei-Ruei Lin, Yun-Ju Chuang, Chih-Hao Lee, Fan-Gang Tseng, Fu-Rong Chen

**Affiliations:** 1 Department of Engineering and System Science, National Tsing Hua University, Hsinchu 30013, Taiwan; jly0428@hotmail.com (W.-R.L.); chlee@mx.nthu.edu.tw (C.-H.L.); fangang@ess.nthu.edu.tw (F.-G.T.); 2 Department of Biomedical Engineering, Ming Chuan University, 5 De Ming Rd., Gui Shan District, Taoyuan City 333, Taiwan; 3 Department of Materials Science and Engineering, City University of Hong Kong, Kowloon, Hong Kong, China

**Keywords:** backscattered electron detector, desktop scanning electron microscope, surface topography

## Abstract

Scanning electron microscopy has been developed for topographic analysis at the nanometer scale. Herein, we present a silicon p-n diode with multi-annular configuration to detect backscattering electrons (BSE) in a homemade desktop scanning electron microscope (SEM). The multi-annular configuration enables the enhancement of the topography contrast of 82.11 nA/μm as compared with the commercial multi-fan-shaped BSE detector of 40.08 nA/μm. Additionally, we integrated it with lateral p-n junction processing and aluminum grid structure to increase the sensitivity and efficiency of the multi-annular BSE detector that gives higher sensitivity of atomic number contrast and better surface topography contrast of BSE images for low-energy detection. The responsivity data also shows that MA-AL and MA p-n detectors have higher gain value than the MA detector does. The standard deviation of measurements is no higher than 1%. These results verify that MA p-n and MA-AL detectors are stable and can function well in SEM for low-energy applications. It is demonstrated that the multi-annular (MA) detectors are well suited for imaging in SEM systems.

## 1. Introduction

The scanning electron microscope (SEM) has been widely applied to study surface topology and the shape of nano-objects in many research fields, ranging from semiconductors [1] to nano-materials [2], life sciences [3,4] and medicine [5]. In an SEM, there are two imaging modes, namely, secondary electron (SE) mode and backscattered electron (BSE) mode. Surface topographic (TOPO) variation is imaged, usually, by collecting SEs, since it results from electron–electron scattering from the surface of the specimen. On the other hand, the contrast of composition (COMPO) in atomic number of the specimen is readily obtained by the BSEs due to its results from an electron–nuclei scattering, and no energy transfer occurs. The SE detector has been usually the standard detector for studying topographic features because the backscattered electron image (BEI) inherently gives poorer spatial resolution than the secondary electron image (SEI).

As compared with the SE detector, the BSE detector has more diverse applications. It is well known that BSE signals can show not only COMPO detail contrast but also TOPO variation [6,7]. Early studies suggest the COMPO and TOPO information can be recorded separately and even optimized using two semi-annular BSE detectors set at, respectively, the high and low take-off angle positions [8,9,10,11,12,13,14,15]. To improve the contrast of surface topography in the BSE mode, a multiple detectors system has been applied [16]. The results show that multiple detectors placed at higher angles (in relation to the *x*-axis) is preferable for topography reconstruction in SEM. However, the multiple detectors system requires a larger space and higher cost.

**Figure 1:**
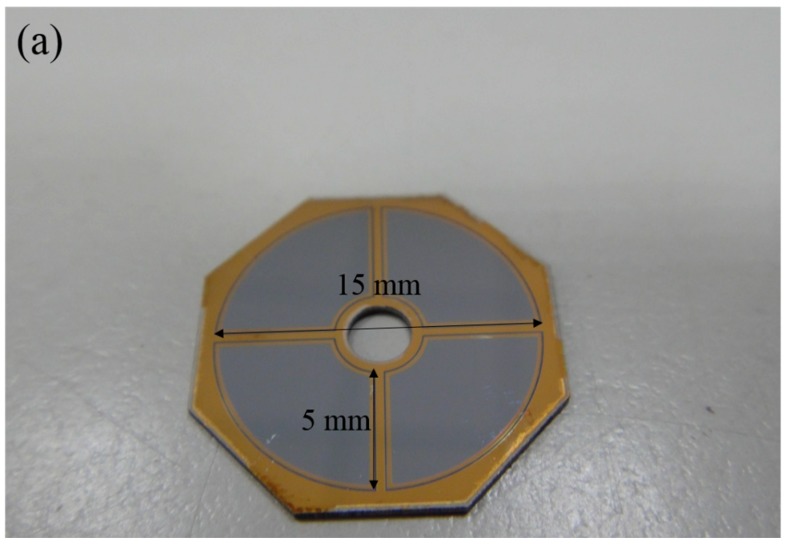

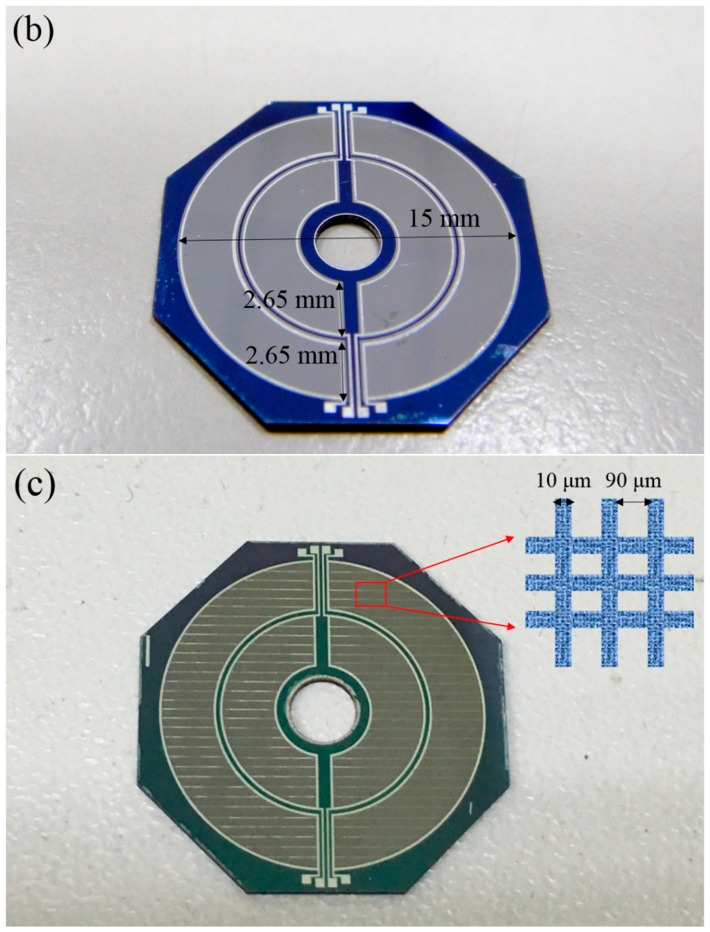
(**a**) The configuration of a commercial (MF) BSE detector with four fan-shaped working areas, (**b**) the configuration of a multi-annular (MA or MA p-n) BSE detector with four semi-ring working areas and (**c**) the configuration of MA-Al BSE detector. The inset shows Al metal grid lines of 10 μm wide and a spacing of 90 μm.

Regarding inspiration from the multiple detectors, in this paper, we modify the traditional commercial MF BSE detector to have multi-annular configuration of detection areas (we called “multi-annular” (MA) detector) which is with the same size as MF BSE detector. The MA BSE detector has a four semi-rings configuration of detection area (two outer rings and two inner rings, as shown in Figure 1b). Outer rings can detect BSEs from low take-off angles in relation to the specimen surface, making the MA detector have better TOPO contrast compared to that of MF detector. Furthermore, it has been proposed that the detector consists of p-type finger structure in n-type silicon as lateral p-n junctions can be an efficient detector for low-energy (e.g., 5 keV–8 keV) imaging in a SEM [17]. In this work, to improve the performance of an MA detector for low-energy imaging in a desktop SEM, we aim to develop a low-cost and easy process to manufacture a MA BSE detector with a lateral p-n junction structure, called MA p-n BSE for low-energy imaging in a desktop SEM. Moreover, we also integrate aluminum grid structures with our MA p-n BSE, termed as MA-Al detector, for a lower-energy electron detection. The aluminum grids can be formed on the surface as an extension of the electrode geometry, which makes lower series resistance of large-area detector increase detector quality factor and efficiency [18]. In this paper, we will demonstrate that our MA p-n and MA-Al detector are well suited for low-energy (5 keV–8 keV) imaging in a homemade desktop SEM.

The design and fabrication of the MA, MA p-n and MA-Al detectors will be first outlined. The experimental results including current-voltage characteristics, topography contrast, and BEI imaging results as well as responsivity information will be shown in the later sections. This paper will also discuss and compare the performance of three BSE detectors, MA, MA p-n and MA-Al in a homemade desktop SEM.

## 2. Design Concept

A BSE detector collects backscattered electrons from the area where the sample interacts with the incident electron beam passing through the central hole of the BSE detector. The traditional commercial BSE detector usually is operated with four fan-shaped (MF) detection areas, as shown in Figure 1a. Here we proposed a multi-annular configuration of detection areas (called a MA detector) which is about the same size as that of the traditional commercial MF BSE detector. We also integrated lateral p-n junctions with the MA BSE (MA p-n) detector, which is the same as the MA except for the implantation method by finger structure. Figure 1b shows the structure of a MA (or MA p-n) BSE detector which consists of multi-annular detection areas. As Figure 1b shows, the outer rings of the homemade BSE detector correspond to low take-off angles in relation to the specimen surface, then can improve topography contrast as compared with the four fan-shaped configuration. For the MA p-n detector, the p-type doping of the finger structure is on the top side and beneath by a p-n junction associated with depletion layers. The total detection areas of the MA (or MA p-n) BSE detector (Figure 1b) is 156.6 mm^2^ which is slightly larger than that of a commercial MF BSE (Figure 1a) detector of 153.6 mm^2^. The solid angle of collection for these MA (or MA p-n) and MF BSE detectors was calculated to be 0.598 and 0.576 sr, respectively [19].

As shown in Figure 1c, a MA p-n BSE detector with metal grids (MA-Al) is patterned with aluminum grids that cover about 6% of the total working area by10-μm-wide Al grid lines. The inset in Figure 1c shows the schematic of the metal grids.

## 3. Fabrication

**Figure 2:**
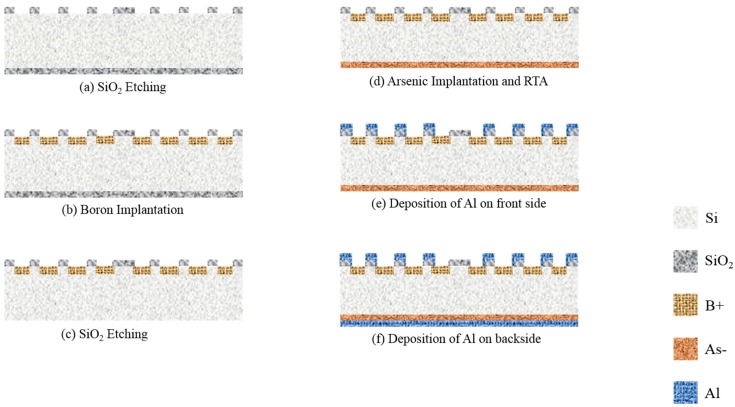
The schematic fabrication processes of the MA BSE detector. (**a**) Front side working areas. A 500 nm-silicon dioxide layer is etched. (**b**) Ion implantation of boron ions on front side. (**c**) A 500 nm-silicon dioxide on backside is etched. (**d**) Backside ion implantation by arsenic ions and then RTA treatment at 950 °C for 60 s. (**e**) A 600 nm-aluminum film is deposited on front side. (**f**) A 600 nm-aluminum layer is deposited on backside.

**Figure 3:**
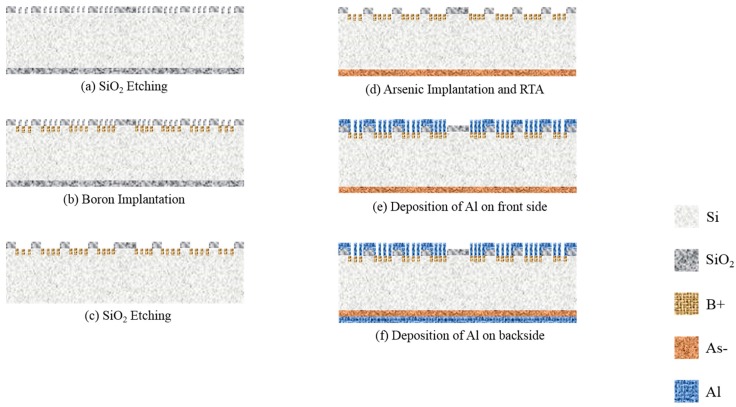
The schematic fabrication processes of the MA-Al BSE detector. (**a**) Front side working areas. A 500 nm-silicon dioxide layer is etched. (**b**) Ion implantation of finger structure by boron ions on front side. (**c**) A 500 nm-silicon dioxide on backside is etched. (**d**) Backside ion implantation by arsenic ions and then RTA treatment at 950 °C for 60 s. (**e**) A 600 nm-aluminum grids film is deposited on front side. (**f**) A 600 nm-aluminum layer is deposited on backside.

A schematic diagram for fabrication processes of the MA BSE detector is shown in Figure 2. The processes for fabrication of MA and MA p-n are quite similar, except the only difference in the fabrication process for the MA and MA p-n devices is that the working area for the MA device is fully doped with boron, while for the MA p-n the doping area for boron is patterned as strips (Figure 3b). The BSE detectors were fabricated on {100} silicon wafer with resistivity of 6000 Ω·cm. The fabrication process was started with a thermal growth of 5000 Å thick SiO_2_ film on both sides of the silicon wafer (Figure 2a). The front side of the wafer was patterned by lithography and etched by buffered oxide etchant (BOE). Then the front side of wafer was ion implanted with boron (Figure 2b). The energy and dose of boron ions were 10 keV and 5 × 10^15^ atoms/cm^2^, respectively. The backside silicon oxide was etched away by BOE again (Figure 2c). Finally, the backside wafer was processed with ion implantation of arsenic (Figure 2d). The energy and dose of As ion were the same as that used in boron implantation. A rapid thermal annealing (RTA) of 950 °C and 60 s was then applied to anneal both sides of the wafer. After RTA treatment, a 6000 Å thick aluminum film was deposited on both sides of the wafer by sputtering (Figure 2e). The metal contacts were then patterned (Figure 2f).

On the other hand, the MA-Al BSE detectors which possibly can enhance surface topography contrast were fabricated with similar processes as stated above for then MA p-n detector. The difference between these two types (MA p-n and MA-Al) of detector is the working area is coated with Al grids. A schematic diagram for the fabrication processes is illustrated in Figure 3a–f.

**Figure 4:**
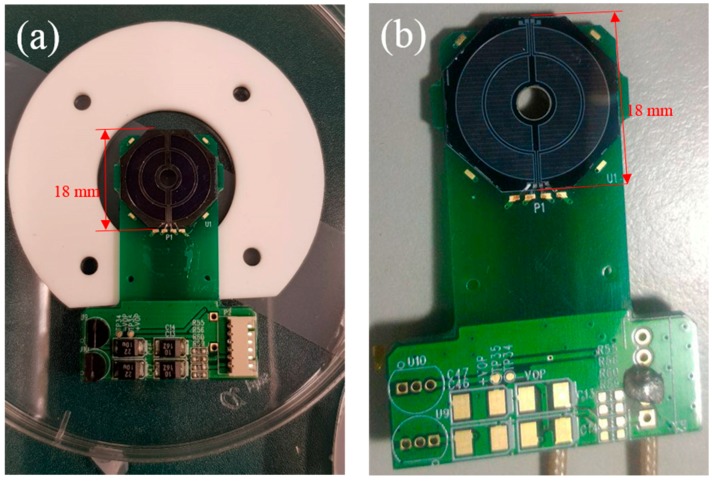
(**a**) The MA BSE detector with wire bonded on PCB, (**b**) The MA-Al BSE detector after wire bonded on PCB.

As shown in Figure 4a,b, after laser dicing, the detectors were wire bonded and mounted on a printed circuit board (PCB).

## 4. Results

**Figure 5:**
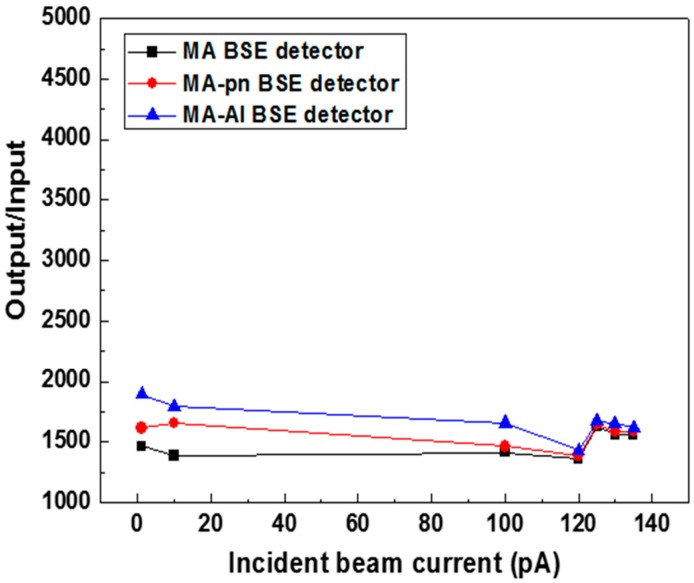
The responsibility gain as a function of different dose rate 15 keV.

To show the MA structure does give a better surface topography contrast as compared with a MF structure, the surface topography (TOPO) contrast was first tested with MF and MA detectors. Then the subtile improvement in electrical performance (I–V curve), effective gain of detectors, sensitivity of atomic number, as well as image contrast for backscattered mode (COMPO) of SEM were tested with three detectors MA, MA p-n and MA-Al BSE detectors. Besides, the stability of our annular detectors has been tested in a desktop SEM. Figure 5 shows the responsibility gain as a function of different dose rate 15 keV.

### 4.1. Surface Topography Contrast

**Figure 6:**
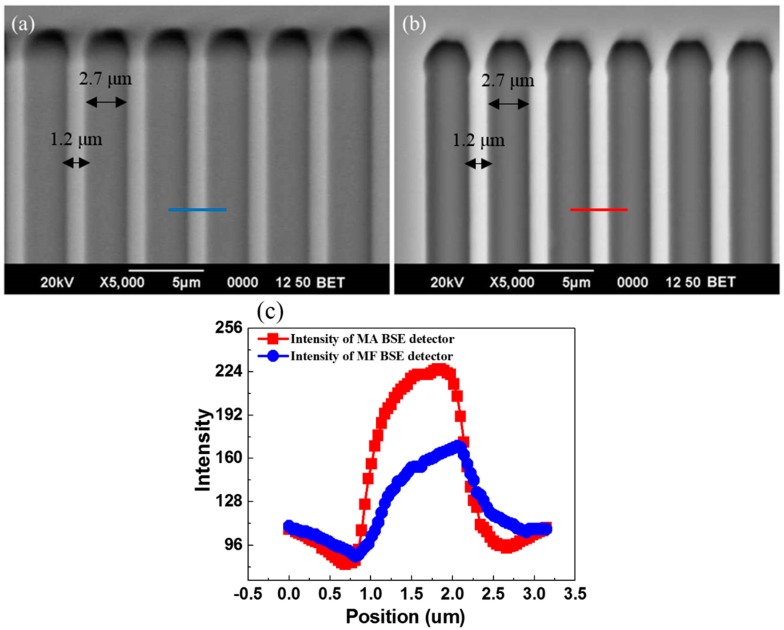
BSEs images of silicon microstructures. The width and depth of silicon micro cavity is 2.7 and 1.0 μm respectively. The spacing between each strip is 1.2 μm. BSE image recorded (**a**) with MF BSE detector and (**b**) with MA BSE detector. (**c**) The intensity profiles across the silicon strip in (**a**,**b**).

In the test of surface topography contrast, two types of BSE detectors, MF and MA BSE detectors were mounted in a SEM (JEOL 6390, Tokyo, Japan). We used BSE images recorded from a commercial MF BSE detector as a bench mark to quantitatively compare with that recorded using our MA BSE detector. The standard sample used for quantification of the TOPO contrast was a silicon patterned microstructures etched by RIE with width of 1.2 μm and depth of 1.0 μm, as shown in Figure 6. The spacing of these strip is 2.7 μm. The topography contrast C is defined as [20],

(1) $$\mathrm{Topography}\;\mathrm{Contrast}=\frac{I_{A}-I_{B}}{H_{A}-H_{B}}$$

where I_A_ and I_B_ are signal currents at the highest and lowest points of the sample, and H_A_ and H_B_ are heights at the corresponding highest and lowest points of the sample. The BSE images recorded using MF and MA are shown in Figure 6a,b, respectively. The topography contrast of our MA BSE detector was determined to be 82.11 nA/μm, which is about twice as good as that of the commercial MF-BSE detector of 40.08 nA/μm. Intensity plots across blue and red lines in Figure 6a,b are shown in Figure 6c. Since outer rings of MA BSE detector locates at lower take-off angles in relation to the specimen surface, it can be properly used for investigation of topography and it is preferable for topography reconstruction in SEM image.

### 4.2. I–V Characteristics

**Figure 7:**
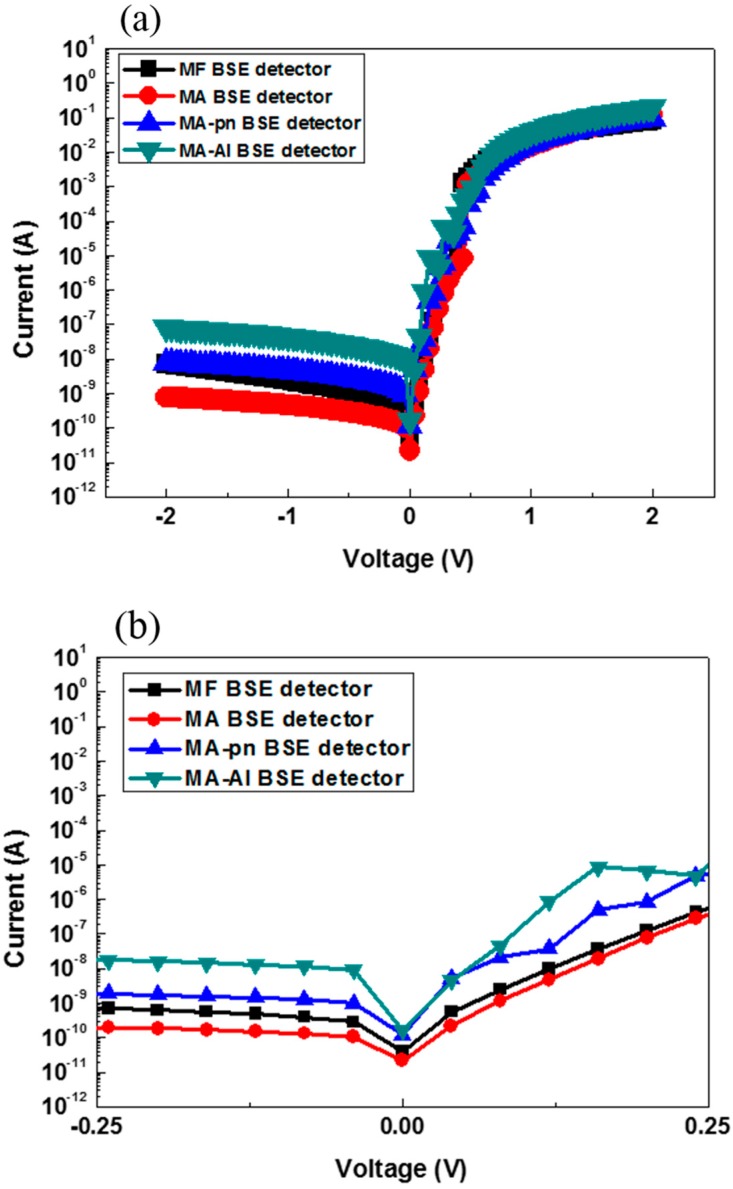
(**a**) Measured I–V characteristic of the BSE detectors without illumination. (**b**) The dark current at zero bias of the BSE detectors.

The basic diode characteristics of electrical performance for the MF, MA, MA p-n and MA-Al detectors were tested by measuring the I-V characteristics. We obtained typical I-V curves of detectors by multi-meter (Keithley 2635B, Tektronix Company Ltd., Shanghai, China) and put the detectors in SEM chamber to make sure that without illumination, as shown in Figure 7. As expected, the I–V characteristics show that these four detectors are ideal and uniform with low dark currents. The dark current of the MA, MA p-n and MA-Al detectors at zero bias are 20 pA, 100 pA and 150 pA, respectively. The dark current of 20 pA is very small and can be negligible in the detection of backscattered electrons. The MA p-n BSE detectors has a higher dark current 100 pA due to aluminum electrode which may directly contact with a non-doping layer of working area of the lateral p-n junctions structure (Figure 2). In addition, the dark current for the aluminum grids structure is 150 pA because that dark current will increase with increasing electrode area (Figure 3). Those can result in an undesirably high dark current.

### 4.3. Effective Gain of Detectors for Electron Energy Lower Than 15 keV

The effective gain G is given by the ratio of detector output current to dose of electron beam (also can be characterized as incident current). To determine the effective gain, we exposed the MA-, MA p-n and MA-Al detectors to direct electron irradiation also in a SEM (JEOL 6390, Tokyo, Japan) and the incident electron beam current was measured by using a Faraday cup.

(2) $$\mathrm{Gain}=\frac{\mathrm{DetectedElectrons}}{\mathrm{IncidentElectrons}}=\frac{I_{\mathrm{output}}}{I_{\mathrm{input}}}$$

**Figure 8:**
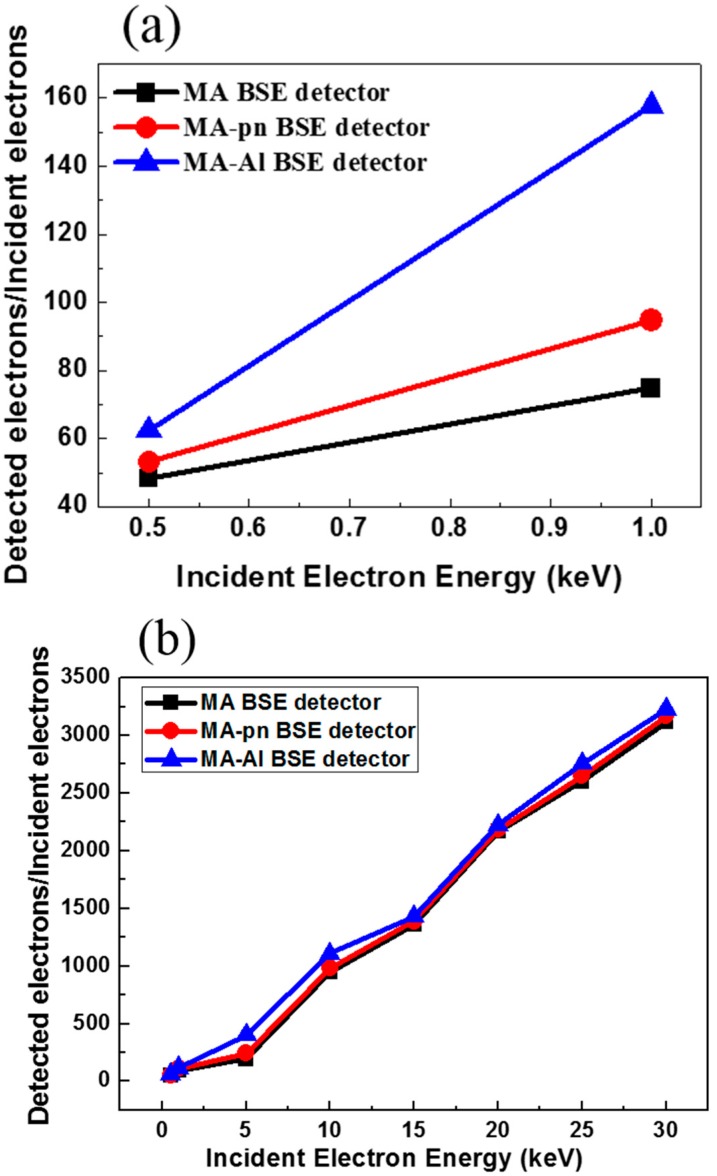
(**a**) The gain measurement for three types of detector at electron energy less than 1 keV. (**b**) Gain measurement for three types of detector at electron energy from 1 keV to 30 keV.

Figure 8a shows that the detector current varies with the incident beam current for electron energy lower than 1 keV, while for the electron energy greater than 1 keV is as shown in Figure 8b. The experimental value of I_input_ is measured from the current of the incident electron beam with a Faraday cup and the I_output_ is measured from the current in our detectors. It shows that the MA-Al detector has better performance for low-energy electron detection. At the electron beam energy of 0.5 keV, the measured electron signal gain of the MA-Al detector has an approximate performance which as compared to the boron-layer silicon photo-diodes, which have been done by other teams [21].

### 4.4. Sensitivity of Atomic Number

To determine the sensitivity of atomic number, we prepared a standard specimen which inlaid five polished materials (Al, Fe, Co, Ni and Ag). Three different, BSE detectors (MA, MA p-n and MA-Al) were mounted in a desktop SEM, EM-100. The sensitivity of atomic number contrast was defined as [20],

(3) $$\mathrm{Sensitivity}\;\mathrm{of}\;\mathrm{Atomic}\;\mathrm{Number}=\frac{I_{A}-I_{B}}{Z_{A}-Z_{B}}$$

where I_A_ and I_B_ are signal currents for a sample of different atomic number; Z_A_ and Z_B_ are corresponding atomic number of different elements.

**Figure 9:**
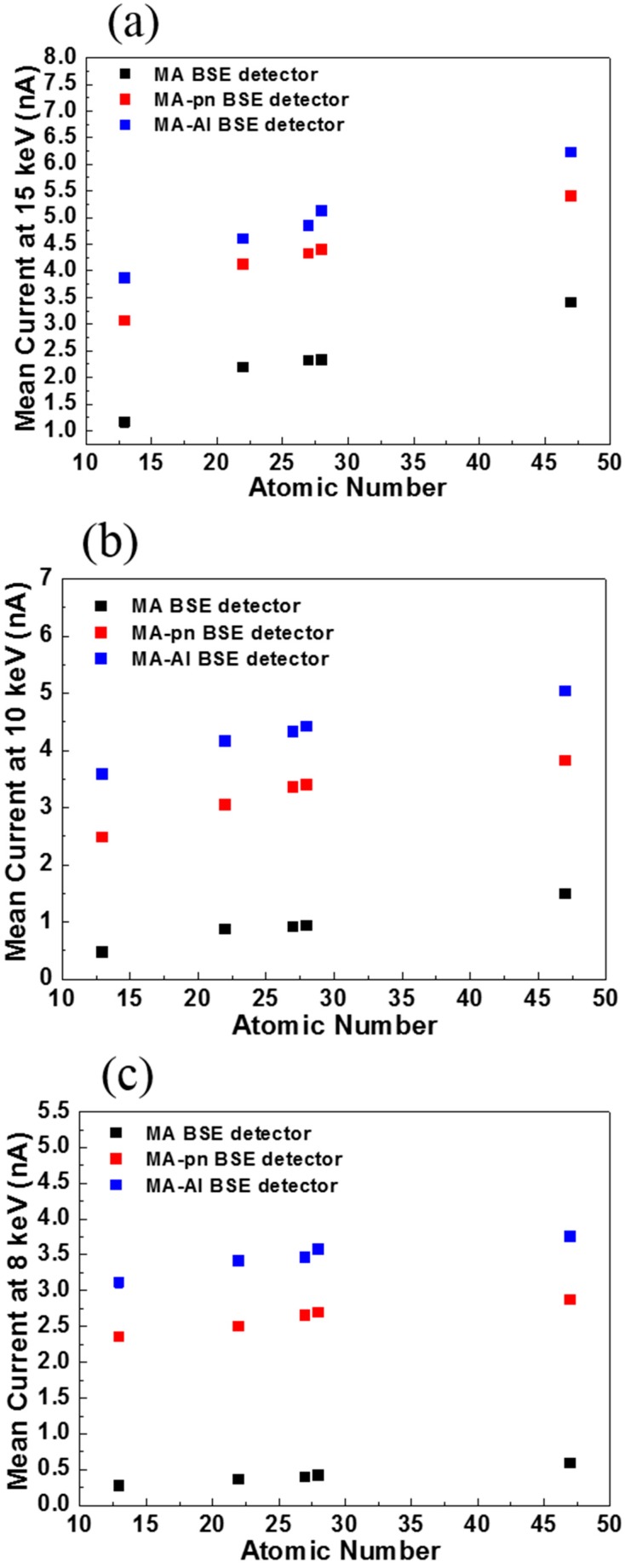
Sensitivity of atomic number for three type detectors at electron energy of (**a**) 15 keV, (**b**) 10 keV and (**c**) 8 keV.

Figure 9a–c show that sensitivity to the atomic number for electron energy at 15 keV, 10 keV and 8 keV, respectively. For electron beam energy at 8 keV, the MA p-n and MA-Al detectors have 64% and 103% enhancement in sensitivity to the atomic number as compared with that of the MA BSE detector.

### 4.5. BEI Contrast

**Figure 10:**
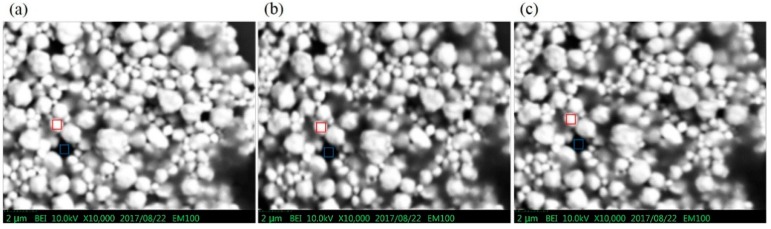
BSE images of the mixed gold particles and polystyrene particles sample taken at 10 keV with (**a**) MA BSE detector, (**b**) MA p-n BSE detector and (**c**) the MA-Al detector.

This experiment was also performed in a desktop SEM (EM-100). The BSE image contrast for three different BSE detectors (MA-, MA p-n and MA-Al) were quantified using a sample of mixed gold and polystyrene particles. The image contrast C can be quantified as [22],

(4) $$C=\frac{\left|I_{\mathrm{Signal}}-I_{\mathrm{Background}}\right|}{\sqrt{\sigma_{\mathrm{Signal}}^{2}+\sigma_{\mathrm{Background}}^{2}}}$$

where I_Signal_ and I_Background_ are the averaged intensity from the circled gold particle and the silicon substrate, respectively, while the σ_Signal_ and the σ_Background_ are the standard deviation of these two parts, respectively. As shown in Figure 10, the contrast of images has been obtained at the electron energy of 10 keV. The mark of red square is for intensity of signal and the mark of blue square is for intensity of background. The image contrasts C for Figure 10a–c are 26.36, 29.61 and 30.61, respectively. According to the image contrast result, the MA-Al BSE detector has about 16% enhancement compared to the original MA BSE detector.

**Figure 11:**
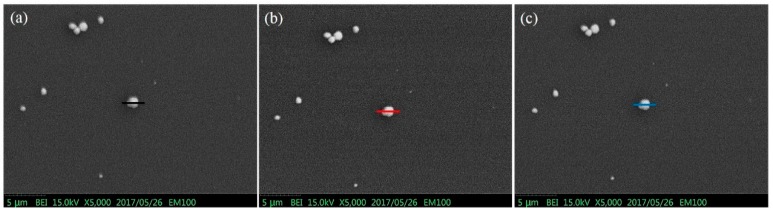

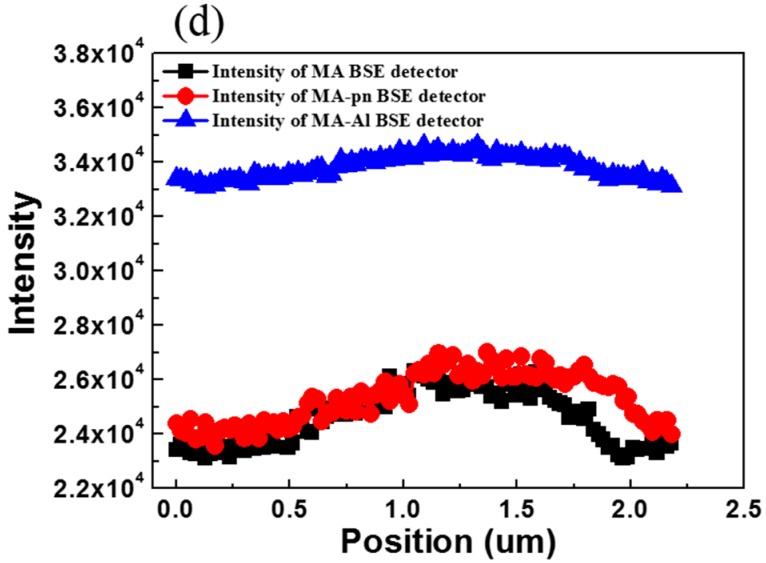
BSE Images of the selected gold particle recorded with (**a**) MA BSE detector, (**b**) MA p-n BSE detector, (**c**) MA-Al detector, (**d**) The intensity profiles across the gold particle of different BSE detectors.

In addition, another sample of pure gold particles is used for the quantitative comparison of different BSE detectors, as shown in Figure 11a–c. Figure 11d shows the intensity profiles across the gold particles. The MA-Al BSE detector also enhanced the contrast of 31% than that of the MA BSE detector. The background intensity for the MA-Al is higher than other detectors because the electrode area will increase with aluminum grids structure which makes the current signal increase.

## 5. Conclusions

In this study, we designed and fabricated a simple processing and low-cost silicon detector with MA configuration of detection area to enhance topography contrast for the application of BSE detectors in desktop SEM. The MA BSE detector can provide better surface topography contrast of 82.11 nA/μm which is compared with the commercial MF BSE detector of 40.08 nA/μm. Moreover, we integrated it with several other special processes to increase the performance of the BSE detector. Without biasing, the detector presented here has a dark current in the order of 150 pA and a detector gain of 62.5 at electron beam energy is 0.5 keV. The modified BSE detector has better sensitivity of atomic number and BEI contrast than the MA BSE detector for low electron energy detection (about 16% enhanced). Further enhancement in detector performance is possible by decreasing the width and pitch of the aluminum grids. Higher capability can be expected and it will become a powerful device in SEM systems.
